# Library of Identification Resources: a FAIR overview of taxonomic keys

**DOI:** 10.3897/BDJ.13.e161726

**Published:** 2025-08-26

**Authors:** Lars G. Willighagen, Eelke Jongejans

**Affiliations:** 1 Department of Ecology, Radboud Institute for Biological and Environmental Sciences, Radboud University, Nijmegen, Netherlands Department of Ecology, Radboud Institute for Biological and Environmental Sciences, Radboud University Nijmegen Netherlands; 2 Department of Animal Ecology, Netherlands Institute for Ecology (NIOO-KNAW), Wageningen, Netherlands Department of Animal Ecology, Netherlands Institute for Ecology (NIOO-KNAW) Wageningen Netherlands

**Keywords:** linked data, FAIR data, taxonomy, identification keys

## Abstract

Biodiversity is declining globally, and ecological research is key to monitor and counteract this decline. Such research requires the taxonomic identification of organisms by both professional and citizen scientists. A complete overview of resources for taxonomic identification is therefore crucial but missing, also posing problems for analysis into gaps in the taxonomic coverage of available identification resources. To create a repository of this kind, we applied the FAIR principles, collected data on identification resources, and created a search engine to find relevant identification resources for a given observation of an organism within this data. So far, the data collection has been strongly biased towards keys for insects of Northwestern Europe, leading to incomplete search results for other, underrepresented taxa and regions, which is then indicated in the interface. Our Library of Identification Resources already contains 2,158 works and is made available as linked data using domain-standard vocabularies including BIBO and Audiovisual Core. To increase the accuracy, size and scope of the repository, processes for entering metadata of identification resources should be streamlined. We discuss how publishers, authors, and libraries could be involved and persuaded to register their own published dichotomous or multi-access keys, species descriptions, field guides, and image- or audio-based identification applications, as inclusion makes their identification resources findable for a larger group of potential users.

## Introduction

Biodiversity is declining globally, affecting a wide range of ecosystem services (IPBES 2019). This decline is driven by habitat loss, pollution, climate change, the spread of alien species, among other factors (Cardoso et al. 2020, Wagner et al. 2021). Ecological research is necessary to monitor biodiversity decline, to understand driving processes like biological invasions, to determine the efficacy and efficiency of management strategies, and to identify areas that most urgently require conservation and management.

The ability and expertise to taxonomically identify species is crucial for ecological research (Elphick 2008, Farnsworth et al. 2013), including derived statistics like species richness, functional diversity, and habitat quality indices. This importance of taxonomic identification applies to both traditional studies by research institutes and for the collection of large-scale biodiversity data by citizen scientists through platforms such as Observation.org and iNaturalist (Campbell et al. 2023, Alfeus et al. 2024). Tools to assist in taxonomic identification also need to be up to date as the local species pool may change over time. For instance, to be able to track the spread of biological invasions, researchers need to be able to recognize newly introduced species in addition to native species, and ideally also alien species that are likely to be introduced in the near future (Douglas et al. 2009, Pyšek et al. 2013).

Resources like identification keys and field guides are pivotal for the taxonomic identification of specimens (Farnsworth et al. 2013, Bowler et al. 2022). It is therefore important for audiences like researchers and citizen scientists to be able to obtain resources most relevant for any given specimen (Godfray 2005). For a reliable identification of an observation or a specimen, an identification resource should capture the extent of taxonomic or morphological variation, as well as variation in characteristics that can occur at the time and location where the organism was observed or collected such as life stage, sex, and behavior. This means that identification resources have a limited taxonomic, geographic and ecological scope which should be described in their metadata for efficient selection of relevant resources. To find suitable resources on large scales, a search engine backed by a complete, machine-readable overview of identification literature is required.

However, there is no complete overview of identification resources that is both machine-readable and reusable. Even overviews of the taxonomic literature in general are rare and require a lot of effort to construct (Cowan 1990, Page 2023). Furthermore, neither the metadata used to describe identification resources, nor formats to describe their contents, are standardised. This does not square with the current push towards open science, including the FAIR Data Principles advocating for Findable, Accessible, Interoperable, and Reusable data (Wilkinson et al. 2016).

To address this, we first defined requirements for making a FAIR overview of identification resources and formulated recommendations for implementing these requirements. Then, we apply these recommendations on a substantial though biased subset of the identification literature to curate an initial overview of identification resources. Finally, an interface and a search engine are created, backed by this overview, that can later be extended with more data.

### Related work

Bibliographies of identification literature exist in various places, from field guides and simple PDFs to forum posts and wiki pages (van Veen 2005, Verheyde 2017). These bibliographies reference relatively few resources, tend to be unstructured, and do not follow the FAIR guidelines. Larger overviews of specific parts of the identification literature have been created (Göllner-Scheiding 1967, Göllner-Scheiding 1969, Göllner-Scheiding 1970a, Göllner-Scheiding 1970b, Göllner-Scheiding 1971a, Göllner-Scheiding 1971b, Gaedike 1976, Gaedike 1981, Gaedike 1986, Gaedike 1988, Sims et al. 1988, Gaedike 1992, Gaedike 1997), but are often not available in a machine-readable format. The few that are (NaDaBa n.d., Storey 2010), are generally not reusable.

An example of an extensive overview published from 1967 to 1971 is the *Bibliographie der Bestimmungstabellen europäischer Insekten (1880–1963)* (Göllner-Scheiding 1967, Göllner-Scheiding 1969, Göllner-Scheiding 1970a, Göllner-Scheiding 1970b, Göllner-Scheiding 1971a, Göllner-Scheiding 1971b), containing approximately 7000 titles of identification keys for various insect taxa. It was followed by a continuation for the period 1964–1995 (Gaedike 1976, Gaedike 1981, Gaedike 1986, Gaedike 1988, Gaedike 1992, Gaedike 1997). Apart from the citations themselves, these bibliographies list additional characteristics of the key, including the type of resource, the taxonomic and geographic scope, and the taxonomic rank to which specimens can be identified. Although for some bibliographies scans are freely available, their contents have, to the authors' knowledge, not been digitized and are therefore not searchable or machine-readable. These limitations also apply to the *Key Works to the Fauna and Flora of the British Isles and North-western Europe*, published in 1988 (Sims et al. 1988), which was intended not as a comprehensive bibliography but as a minimal set of titles to cover all species in the British flora and fauna.

Digital overviews do also exist, but do not adhere to all FAIR guidelines. The website BioInfo, created in 1998 and last updated in 2024, contains a curated bibliography of 9,534 references, with additional metadata on the applicability of the key and requirements put on the observer, e.g. the use of a stereo microscope or the preparation of genitalia (Storey 2010). The database is licensed under an open license (CC BY-NC-SA 4.0), but does not offer downloads or an index, instead directing users to Google Search. Another digital overview, NADABA, was started in 2022 to "catalog the tools used to identify and locate fauna and flora around the world" (NaDaBa n.d.) and contains 661 resources. Contrary to BioInfo, NADABA does have an index, including basic metadata search. However, it contains less information on the applicability of the tools in its database, and does not specify their taxonomic scope further than general categories. Additionally, the database is not made available for export, and no copyright license is specified.

## Material and methods

We interpreted the FAIR principles for the purpose of creating a new, open catalog of identification resources (Suppl. material 1). These recommendations first of all apply to the metadata of identification resources. This is because the data, being the identification resources themselves, are too heterogeneous for a single set of recommendations.

### Data collection

Based on the principles in Suppl. material 1, we created a catalog named the *Library of Identification Resources* (LoIR) available at https://identification-resources.github.io/. Any piece of media or computer program that could assist in the taxonomic identification of organisms was considered to be an identification resource, and therefore relevant as an entry to the catalog. In particular, this included traditional dichotomous identification keys and (digital) multi-access matrix keys (Walter and Winterton 2007), lists of detailed (diagnostic) species descriptions, galleries of images, regional checklists, and image- or audio-based automatic recognition applications. Additionally, resources consisting of a collection of links or references were added when appropriate, as well as supplements and errata, if they were published separately.

So far, entries have been added to the catalog in an opportunistic fashion, mainly focusing on identification resources useful for insect species in Northwestern Europe, meaning the available literature from other taxa and regions is currently underrepresented. Further entries were added through backward citation search (snowballing) and by adding national or international series of handbooks such as the *Royal Entomological Society Handbooks* (United Kingdom), *Faune de France* (France), *Nationalnyckeln* (Sweden), *Danmarks Fauna* (Denmark), and *Fauna Ibérica* (Portugal & Spain) and some of the larger collections of digital resources such as those by the Naturalis Biodiversity Center (Netherlands).

**Figure 1.**
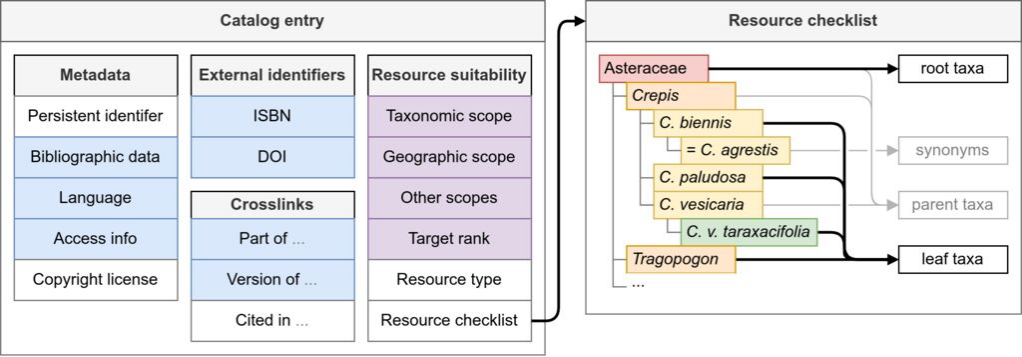
**Schematic overview of the data model of the Library of Identification Resources, showing how the metadata of identification resources and associated checklists are represented following FAIR principles.** The catalog entry proper, shown in the left box, mostly consists of bibliographical data expressed using the BIBO ontology (in blue) and domain-specific data using the AC ontology (in purple). Optionally, a taxonomic checklist of the resource can be associated with a catalog entry, indicating which taxa are included. For this data, a simplified example is shown in the right box, where red, orange, yellow, and green represent different taxonomic ranks, respectively family, genus, species, and subspecies.

Catalog entries consisted of general metadata modeled using the Bibliographic Ontology (BIBO, D'Arcus and Giasson 2013), including a persistent identifier that can be resolved through purl.org, bibliographic metadata, the language of the resource, information on how to access the resource online, and if available the copyright license, and additional external identifiers (Fig. 1, blue boxes). Wherever possible, different editions and prints of books as well as different versions of digital resources were added as separate entries in the catalog. This made it possible to collect and display accurate bibliographic references, and to reflect any changes in the taxonomic scope of the resource. Versions, editions and translations were linked together in the data model using BIBO (Fig. 1).

Resource suitability was recorded f or every resource in the catalog using terms aligned with Audiovisual Core (AC, Baskauf et al. 2022) (Fig. 1, purple boxes), in accordance with FAIR principle F2 (Suppl. material 1). This primarily consisted of a taxonomic scope, restricting the resource to some part of the tree of life, and the geographic scope (Koch et al. 2022), which can range from the entire biosphere to a biogeographic (sub)realm, continent, country, or a narrower geographical region (see examples in Table 1). Resources may in some cases apply to a narrower set of species than specified by this combination of scopes (e.g. Ellis 2001), such as only aquatic species or only common species, which was then recorded in a simple text field. Secondly, the applicability of a resource also depends on characteristics of the individuals (such as their life stage and sex) and whether the evidence of the individual itself or indirect evidence (like an egg case, a nest, or a track) is being identified. For these characteristics, AC and the GBIF vocabularies were used where possible. Finally, while the intention often is to identify to species level, keys to higher taxa are useful as well (Chessman 1995). To account for this, the taxonomic target ranks of the identification resources were also recorded. General characteristics of a key can optionally be recorded as well, including whether it is an artificial or systematic key, and whether it requires the use of a microscope.

Modeling the scope of identification resources makes it possible to determine whether they are applicable for a situation in certain cases, but not in others. Keys might still be reliable (or at least useful) outside of their explicit geographical or implicit temporal scope, and as stated before, the taxonomic scope may have been narrowed further based on subjective characteristics (e.g. "common species" or "species similar to *Halyomorpha halys*", see Table 1).

To determine the applicability of such resources in spite of these challenges, checklists were composed containing all species and other taxa that were included in a resource. These resource checklists contain the starting point(s) of the key or resource (root taxa), the endpoints (leaf taxa, i.e. without child taxa), the hierarchical structure (parent taxa), and synonyms (Fig. 1, right container). If different sections of the resource covered different sets of species, the sections were included with separate checklists. For example, a resource with a key to the species of Muscidae (family including house flies) may include a key to the families of Diptera (e.g. d'Assis-Fonseca 1968). Similarly, a resource with two keys to the species of Pentatomoidea (superfamily of shield bugs), respectively for adults and nymphs, may include different sets of species because the nymphs of some species are undescribed (e.g. Puchkov 1961).

### Search engine

**Figure 2.**
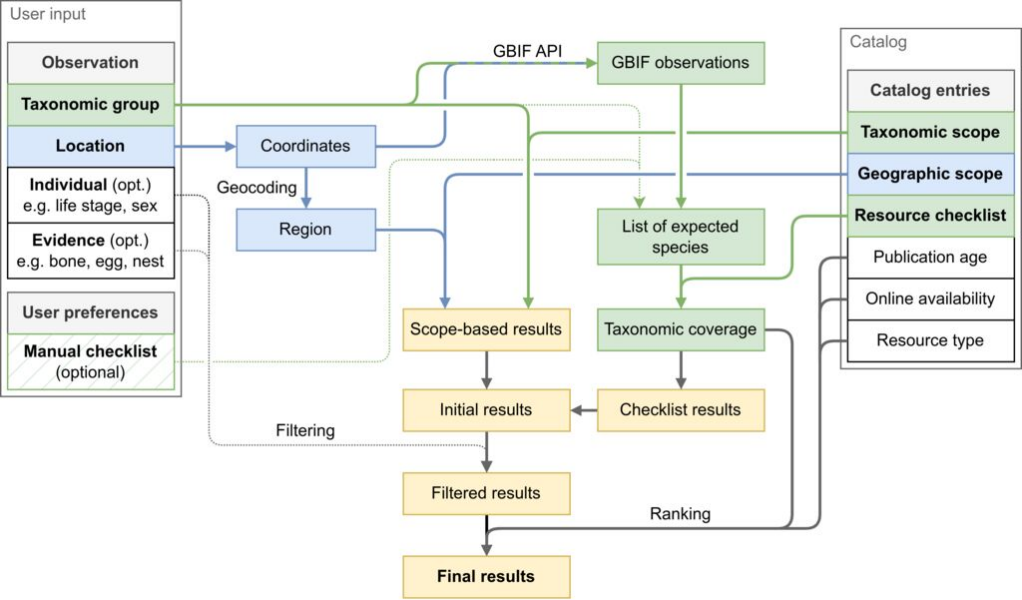
**Schematic overview of the algorithm of the search engine of the Library of Identification Resources, showing how a ranked list of relevant identification resources is derived from a user query.** In the search engine, user input (left) is matched with catalog entries (right) in several, domain-specific aspects, including matching of taxonomic data (in green) and geographic data (in blue) to generate a list of search results (yellow). These results are then optionally ("opt.") filtered by user configuration (marked with thin, dotted lines and hatched background colors), and ranked on various metrics. An example of a query and accompanying search results is shown in Suppl. material 4.

To use the LoIR data in practice, we created a search engine (Fig. 2). Since the search engine relies on the domain-specific data model described above, it is currently limited to the curated database of the LoIR and therefore inherits its biases. As a search query, it takes information about the organism for which an identification resource is needed (Fig. 2, leftmost container labeled "User input"). The initial information entered includes a coarse taxonomic identification to a certain group, which may be omitted to indicate the identification is simply "Biota", and a location. Optionally, characteristics of the individual (such as life stage or sex) as well as the type of observation (such as that of an egg case, track, nest or set of bones) can be specified.

Then, it compares the information entered to the entries of the LoIR catalog (Fig. 2, rightmost container labeled "Catalog") in two distinct ways. First, the location of the occurrence is resolved to a set of coordinates ("reverse geocoding") using the iNaturalist API (iNaturalist n.d.). From these coordinates, a set of encompassing regions is determined ("geocoding"), using the iNaturalist API as well. These are filtered and compared to the geographic scope of the catalog entries (Fig. 2 , blue boxes). Simultaneously, the taxonomic group of the organism is compared to the taxonomic scope of the LoIR entries to get a list of results based on the scope of the resource (Fig. 2 , green boxes). Second, a checklist of expected species is created from the taxonomic group and the location of the occurrence using the GBIF API (Global Biodiversity Information Facility n.d.). This list can optionally be overridden by manually specifying a checklist, which gets filtered according to the same taxonomic group. This checklist of expected species is then compared to the checklists associated with the different resources in the catalog to get another list of results (Fig. 2, green boxes).

The results of both comparisons are merged to form a set of initial results, which can optionally be filtered based on other characteristics of the observation (Fig. 2 , yellow boxes). Finally, the results are ranked using several characteristics: the specificity of the taxonomic scope, the taxonomic coverage (based on the checklists), the age of the publication, whether it is available online, and the estimated amount of distinguishing power based on the type of resource. This ranking formula is explained in detail in Suppl. material 2, and its three main components are indicated for each search result in the interface. The age of the publication is particularly relevant as older resources may not cover introduced species, while newer resources may not cover locally extinct species, placing a temporal restriction on which observations and specimens can be reliably identified. Additionally, older publications may use outdated taxonomy, terminology, and identifying characteristics.

## Results

**Figure 3.**
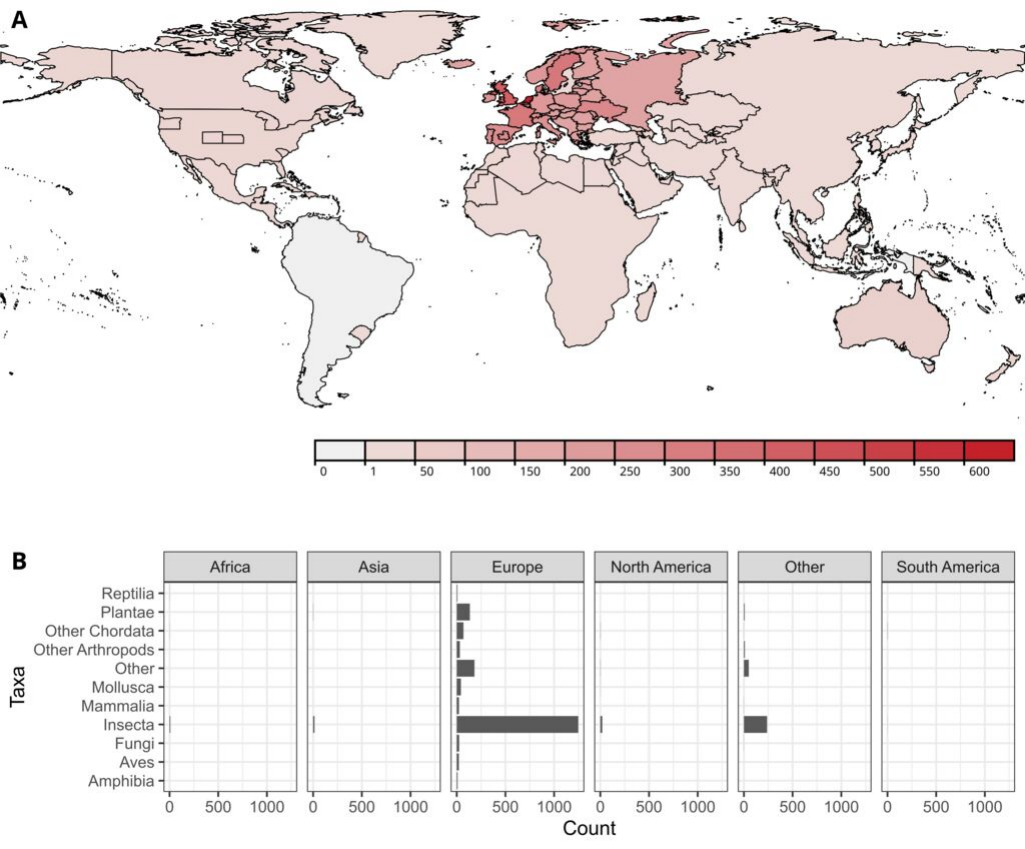
**Geographical and taxonomic focus of the resources currently included in the Library of Identification Resources.** (A) Choropleth of the geographic scopes of resources in the catalog. 460 publications with a geographic scope that cannot be expressed in administrative borders were omitted. (B) Breakdown of publications by the taxonomic group and continent. Publications spanning multiple continents and/or multiple taxonomic groups are counted for the category "Other".

At the time of writing, the LoIR catalog contains 2,158 entries, of which 1,765 unique works and 390 translations or different versions or editions of unique works. The entries primarily (70%) consist of dichotomous keys to species (Suppl. material 3 b). The catalog contains metadata for both print and online media, with about 647 entries being online-only or primarily online publications (Suppl. material 3 d). Though not all works that are associated with metadata entries are publicly available, 1,166 entries have a full-text URL, and of those 405 have an open license or are in the public domain (Suppl. material 3 c). In the current version of the catalog, most of the entries concern insects (1,529), followed by resources treating multiple groups (234) and plants (145). In terms of geographic scope, most entries treat parts of Europe (1,795) followed by resources treating multiple continents (309). Out of the total of 2,158 entries, 1,248 concern insects in Europe specifically, compared to 20 for North America, 14 for Asia, 8 for Africa, and only 1 for South America (Fig. 3).

To determine the applicability of these resources, checklists were composed containing all species and other taxa that were included in a resource for 690 of the resources in the catalog. These checklists were converted to tabular Darwin Core (DwC, Darwin Core Maintenance Group 2023) files containing information on the taxa, their hierarchy, and any synonymy. The checklists have a mean of 94 leaf taxa (median: 25), a mean of 17 synonyms (median: 0; 67.7% of the resources do not specify any synonyms), and a mean of 27 parent taxa (median: 3). Where possible, the scientific names of these taxa were linked to the Global Biodiversity Information Facility (GBIF) Backbone Taxonomy (GBIF Secretariat 2023) and the Catalogue of Life (COL, Bánki et al. 2024) using the Global Names Verifier tool (Mozzherin 2024). Of the leaf taxa, 96.5% are matched to a taxon in the GBIF Backbone Taxonomy, and 85.7% are matched to a taxon in the COL. Among synonyms this is 82.0% for GBIF and 61.0% for COL.

The search engine of LoIR provides lists of the most relevant identification resources for a given specimen or observation. To illustrate the use of the search engine, we show the results when we searched for the soldier fly genus *Stratiomys*, while indicating that the observation took place in Switzerland (Suppl. material 4). The search engine first determines a checklist of expected species based on GBIF observations, in this case consisting of *S. chamaeleon*, *S. concinna*, *S. longicornis*, *S. potamida*, and *S. singularior*, which it uses to determine taxonomic applicability. In this example, the LoIR search engine listed the top 10 hits: 1 matrix key, 4 dichotomous keys, 3 checklists, 1 set of diagnostic descriptions, and 1 collection of other resources. The hits are ordered by a compound ranking score shown to the right, which can be clicked on to reveal its calculation. For instance, the top hit in this Swiss *Stratiomys* search had an overall score of 0.85, which was the product of three factors: taxonomic and geographic applicability (0.87), usability (1.00) and recency (0.97). This matrix key thus received only a small penalty for being slightly older (2016), but a more extensive ‘applicability’ penalty for missing an Alpine species, *Stratiomys concinna*. As the checklist of this particular matrix key is included in LoIR, the percentage of included species (here: 4 of the locally expected 5 species: 80%; or when scaled by the number of observations: 93%) is provided below the record of this matrix key (see Suppl. material 4). In contrast, the sixth hit, an Italian checklist of soldier flies, contains all five species, scores maximally (1.00) for taxonomic and geographic applicability, but only 0.70 for usability (a penalty for being ‘only’ a checklist, see Suppl. material 2) and 0.97 for recency (2013), resulting in an overall score of 0.68. By making the weighing of the ranking of the found resources comprehensible, users can make informed decisions on which identification resources to use first. The second hit, the first volume of a multivolume book treating all Stratiomyidae of Europe, is expected to include all 5 expected species as well, though there is no available checklist associated with the resource. Because of this, it has high taxonomic and geographic applicability (0.97) but as it is not available online and was published in 1982, it has low usability (0.90) and recency (0.88) scores.

## Discussion

With our Library of Identification Resources (LoIR) we have introduced a functional, FAIR registry of identification resources. Already, LoIR can assist researchers interested in identifying insects in Northwestern Europe by providing an overview of the most relevant identification resources with links and PDFs of online or digitized resources where available. However, it should be noted that the registry is not, and is unlikely to ever be, an exhaustive list of identification resources. Currently several taxa and regions are severely underrepresented, which is acknowledged on the LoIR website. Therefore, a query that returns no results does not imply the non-existence of relevant literature. This message is clearly communicated on the website, while at the same time inviting users to provide information on identification resources that are missing in the catalog. At the moment, LoIR contains 2,158 entries and that number keeps growing. Developments in the findability and interoperability of resources can further support the growth of the project to a broader scope.

### Adoption of better standards for identification keys would improve the ease of data reuse

The reuse of data on identification resources and their contents, including for the creation of a searchable library, is hampered by current standards being incomplete and not widely adopted. To make such metadata and data fully compliant with FAIR guidelines, certain changes are needed to the applicable standards and vocabularies, particularly those from Biodiversity Information Standards (TDWG) such as Audiovisual Core (AC), DwC, and Structured Descriptive Data (SDD, Hagedorn et al. 2005).

For the metadata included in LoIR, AC is a reasonably suitable vocabulary. However, it still misses terms for some of the metadata collected for LoIR (Suppl. material 5). AC does not yet contain subtypes for different types of identification resources, only the generic type "Identification Key", meaning part of the data in LoIR cannot be represented. Additionally, at the moment AC only applies to digital media or physical media encoding audiovisual data, but not to analog content such as printed books or journal articles. Finally, terms for scope, like "life stage" and "sex", do exist but are intended to describe the contents of images and videos, not necessarily the scope of an identification key. Future updates to AC could add more subtypes of identification keys and physical media, and clarify the definitions of terms for the different scopes, resolving the limitations that we encountered when creating LoIR.

The content of the identification resources should also be made available in an interoperable format when possible and feasible, to enable the functional archival of resources, to develop common tooling and infrastructure (Hagedorn et al. 2005), and to facilitate and streamline the different efforts to create software for authoring and using dichotomous keys and matrix keys (Dallwitz 1980, Vignes-Lebbe et al. 2016, Naturalis Biodiversity Center 2020, Kerner et al. 2021, Koch et al. 2022). Such an interoperable format could be found in SDD (Vignes-Lebbe et al. 2016, Kerner et al. 2021), provided it is updated to align with AC for metadata and DwC for taxonomy. The adoption of interoperable data formats also depends on the publishers and authors, as the willingness and capability of following the FAIR guidelines differs among commercial publishers, large scientific organizations, and citizen scientists. The potential for reuse of interoperable data further depends on the availability of the copyright license and provenance metadata of the resource.

Another aspect of both the metadata and data of identification resources that should be considered in the discussion on their interoperability is the scientific nomenclature and the associated taxonomy. For the taxonomic data within a resource, the DwC vocabulary is suitable. To enable the comparison of taxonomic data across resources, and to follow principle I3 of the FAIR guidelines (Suppl. material 1), these scientific names should also be linked to taxonomic databases. However, there is no singular database with persistent, unique identifiers for taxa that is complete enough to be usable for the purposes of LoIR. Specific databases exist for separate groups of organisms, e.g. Index Fungorum (fungi), International Plant Names Index (plants and fungi) and the Index to Organism Names (mostly animals) and ZooBank.org (animals). However, these indices often have multiple identifiers for the same name, within the same index or across multiple indices, and offer no or limited metadata and taxonomy (Page 2023). GBIF also assigns identifiers in its Backbone Taxonomy, does have metadata and taxonomic info, and has fewer duplicates than the aforementioned databases, but explicitly excludes taxa with non-Linnean ranks such as superfamilies, subfamilies, and tribes. The Catalogue of Life offers persistent identifiers but is at the moment not complete enough for applications such as this one, missing 14.3% of leaf taxa compared to 3.5% in GBIF. This poses challenges for interoperability, firstly when comparing checklists, where any missing identifiers could lead to false negatives, and secondly when constructing dynamic checklists based on GBIF data, where a consistent taxonomical hierarchy is essential.

### Accelerating the growth of LoIR would benefit from cooperating publishers

To expand the scope of our Library of Identification Resources beyond the current geographical and taxonomic focus and to represent other regions and taxonomic groups better, existing identification resources need to be entered into the catalog on a larger scale. To facilitate the findability of identification resources, the existing guidelines for the publication of identification keys (Penev et al. 2012) should be amended to specify the type of metadata that should be made available with all keys. Ideally, these guidelines should also recommend making both the metadata and the key itself available in a machine-readable format that allows conversion to SDD. To increase the rate of indexing in a catalog such as LoIR, publishers and journal editors should be encouraged to register published identification resources themselves, as is done for zoological taxonomic acts in ZooBank (Page 2023). Publishers like Pensoft already embrace FAIR principles like machine-readability and reusability (Penev 2017), and might be convinced to do so for identification resources as well. If the works they publish have machine-readable metadata and are included in a central registry, it could improve their visibility. For works from uninterested or dissolved publishers, libraries could collect metadata instead, furthering their mission of making information available to users. Enthusiastic (citizen) scientists and regional taxonomy experts who already compile lists of useful identification resources on forums and websites to share with colleagues, friends, and others (van Veen 2005, Verheyde 2017) could perhaps be persuaded to do so in a more structured fashion on a more central platform, such as LoIR. Already, a user of the iNaturalist platform submitted their own identification key for the *Lygaeus* species of North America for addition to the LoIR catalog (Pirrello 2025), which would not have been found through regular pathways of literature search. For online identification resources such as personal websites, having a simple user-interface to submit entries to LoIR could improve indexing bandwidth. The advantage of this is that registering a publication in a catalog like LoIR increases the exposure of new and existing identification resources to a larger group of potential users, benefiting both publishers and authors. Additional infrastructure to streamline this process is in development.

### Automatic search results remain provisional without expert review

Although the search engine does not claim to be definitive or exhaustive, its results can still be improved by involving experts. Certain aspects of identification resources are not easily determined by those not yet familiar with the resource, including the clarity of descriptions and the correctness of identifying characteristics and the taxonomy, and as such are not specified in the main catalog. We plan to implement a feature allowing people to leave reviews of identification resources, which can be used to adjust the ranking of search results.

Additional potential for improvements can be found in the comparison between species lists as a measure of taxonomic applicability. This comparison is an attempt to estimate the morphological variation that an identification resource covers and find overlaps with the expected morphology of the observed specimen. This method cannot always be used for resources distinguishing only higher taxa, as these often do not list the species considered in the production of the resource. Additionally, just as there is geographic variation in morphology within higher taxa, there is also geographic variation in morphology within species (Lecocq et al. 2015, Falk 2024). Ultimately, this means that the species coverage is not a perfect method to determine the reliability of an identification resource. For ecological research, it is therefore important that resources intended for different geographical regions are not used without validating their reliability, even if the species lists match. This check could be included in the aforementioned expert reviews.

### LoIR enables analysis of patterns in identification literature

The usefulness of a FAIR overview of identification resources extends beyond supplementing and spreading taxonomic expertise. A machine-readable, systematic overview where resource checklists are linked to taxonomic databases can be used to identify gaps in the literature, when the assumption can be made that the catalog lists all relevant taxonomic keys of a specific region. For example, recently introduced or potentially invasive alien species may not be included in identification resources, or only included at a later date, with potential consequences for early detection and efforts to monitor invasions (Douglas et al. 2009). Similarly, groups of species for which few or no identification resources are available in a specific region and language, either because they are inherently difficult to identify or due to the (local) scarcity of taxonomic expertise or interest in a taxonomic group, may be underrepresented in citizen science data (Campbell et al. 2023, Alfeus et al. 2024). Identifying such groups can help to expose systemic barriers for taxonomic and ecological work on specific taxa and regions. Once such underrepresented taxa are identified, the participation of expert taxonomists is essential to gather new taxonomic knowledge, create new identification resources, and overcome these barriers.

We are planning to perform an analysis on the species lists of identification resources cataloged in LoIR to determine which species that are not native to the Netherlands, are included in identification resources used in the Netherlands. Using these data, we can test for a potential effect on detection and monitoring caused by the inclusion or exclusion of alien species in local identification resources. For example, we can compare changes over time in the list of invasive plant species in the 24 different editions of Heukels' Flora (Duistermaat 2020) to contemporary records of plant biodiversity.

## Conclusions

To monitor and counteract biodiversity loss, ecological research is required, which in turn requires the continued development of taxonomic expertise. To support this, we interpreted the FAIR principles for an overview of resources for the identification of organisms and implemented it with the Library of Identification Resource (LoIR), which is available online. Though not exhaustive, LoIR is already useful for finding the most applicable key or other resource for the identification of many groups of insects in Northwestern Europe. Furthermore, it can be extended to a global scope and to organisms across the tree of life, especially if publishers, authors, and taxonomy experts are involved in registering their own works. It is also useful for identifying shortcomings in the existing corpus of identification resources.

## Data availability

Data are available as linked data and as a set of CSV files at https://doi.org/10.5281/zenodo.15552611.

## Code availability

Code for parsing and managing the data is available at https://doi.org/10.5281/zenodo.8208214. Code for the site is available at https://github.com/identification-resources/identification-resources.github.io and code for the search engine is available at https://github.com/identification-resources/find-resources.

## Supplementary Material

51C1BE1C-F884-5E14-91F4-7DD687E49BBA 10.3897/BDJ.13.e161726.suppl1 Supplementary material 1 Table S1 Data type table Brief description Recommendations for making a catalog of identification resources with FAIR metadata. File: oo_1345892.xlsx https://binary.pensoft.net/file/1345892 Lars G. Willighagen, Eelke Jongejans

8883CACC-0803-5A82-A89E-CF79787854C4 10.3897/BDJ.13.e161726.suppl2 Supplementary material 2 Table S2 Data type table Brief description Factors influencing the ranking of results in the search engine of the Library of Identification Resources (LoIR). Scores for the various factors are multiplied to calculate the final search score. File: oo_1345911.xlsx https://binary.pensoft.net/file/1345911 Lars G. Willighagen, Eelke Jongejans

F08F868D-0F88-5E9C-B914-4BB70127F904 10.3897/BDJ.13.e161726.suppl3 Supplementary material 3 Figure S1 Data type image Brief description Summary statistics of the resources in the catalog of the Library of Identification Resources. (A–D) Number of resources in different categories, respectively the main language(s), main resource type(s), main medium, and availability. File: oo_1388482.png https://binary.pensoft.net/file/1388482 Lars G. Willighagen, Eelke Jongejans

38E345F1-2369-5329-A5D1-CF0ABC2F2D20 10.3897/BDJ.13.e161726.suppl4 Supplementary material 4 Figure S2 Data type image Brief description Example results from the search engine of the Library of Identification Resources. The search query consists of "*Stratiomys* Geoffroy, 1762", a genus of weapon flies, in Switzerland. The left section of each result shows the identifier and pictograms for the type of resources, the middle section shows bibliographic information, and the right section shows the percentage of possible species that are included in the resources. File: oo_1345294.png https://binary.pensoft.net/file/1345294 Lars G. Willighagen, Eelke Jongejans

4608B7CE-4B24-55FF-B4C0-5AC7995381C9 10.3897/BDJ.13.e161726.suppl5 Supplementary material 5 Table S3 Data type table Brief description Concepts used to describe identification resources in the Library of Identification Resources (LoIR) and related (meta)data formats. Audiovisual Core (AC) is primarily intended to describe audiovisual media, and as such do not apply to printed resources. Additionally the concepts marked with an asterisk (*) are not intended to encode scope in AC. Structured Descriptive Data (SDD), Clavis, and Delta are primarily intended as data formats, and as such include less metadata. File: oo_1345912.xlsx https://binary.pensoft.net/file/1345912 Lars G. Willighagen, Eelke Jongejans

## Figures and Tables

**Table 1.** Four examples of the modeling of the scopes of identification resources (Willighagen 2022). The identifiers (e.g. "B460") can be resolved to the corresponding resource in the Library of Identification Resources (e.g. https://purl.org/identification-resources/catalog/B460).

**Identifier and title of resource**	**Taxonomic scope**	**Geographic scope**	**Restriction**	**Target rank**
**B460**: A revision of the world Embolemidae (HymenopteraChrysidoidea)	Embolemidae	—	—	species
**B1**: Identification key to the European species of the bee genus *Nomada* Scopoli, 1770 (Hymenoptera: Apidae), including 23 new species	* Nomada *	Europe	—	species
**B81**: Key to some European species of Xylomyidae	Xylomyidae	Europe	"some species"	species
**B63**: MOSCHweb - Interactive key to the genera of the Palaearctic Tachinidae (Insecta: Diptera)	Tachinidae	Palaearctic realm	—	genus
