# Supplementary figures and images for: Library of Identification Resources: a FAIR overview of taxonomic keys
Source: Biodivers Data J. 2025 Aug 26;13:e161726. doi: 10.3897/BDJ.13.e161726 (PMC12405937; doi:10.3897/BDJ.13.e161726)

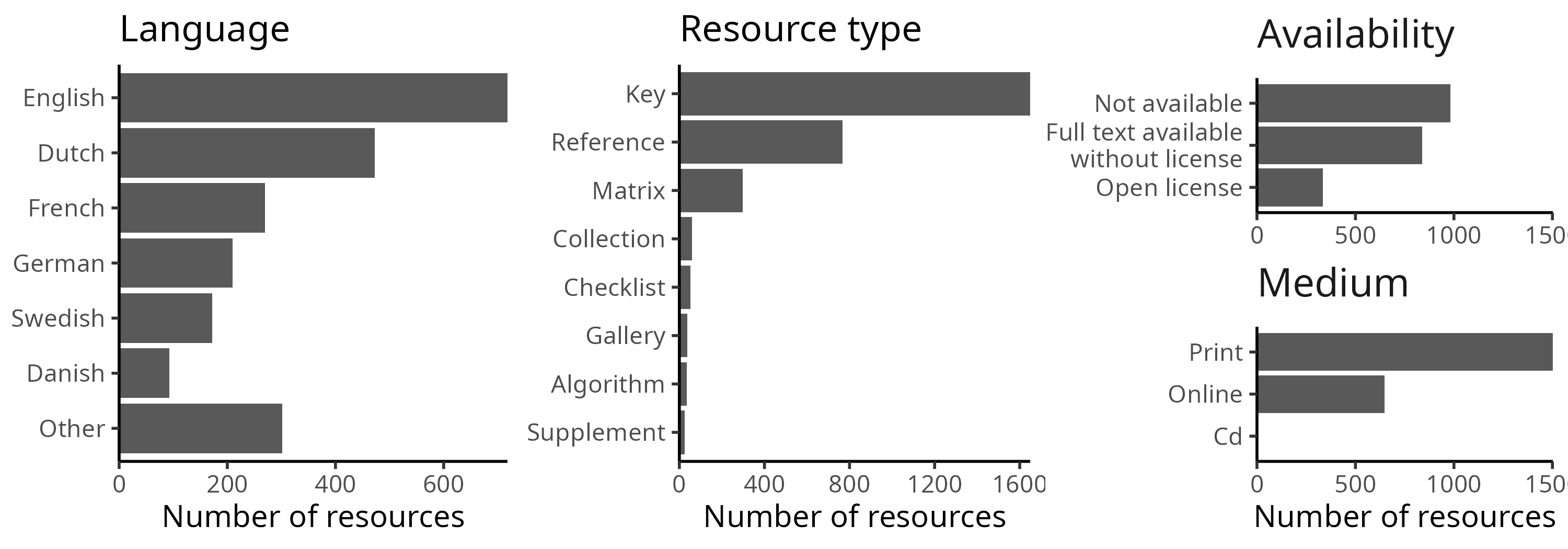

Supplement: Supplementary material 3 — Figure S1 [file bdj-13-e161726-s003.png]

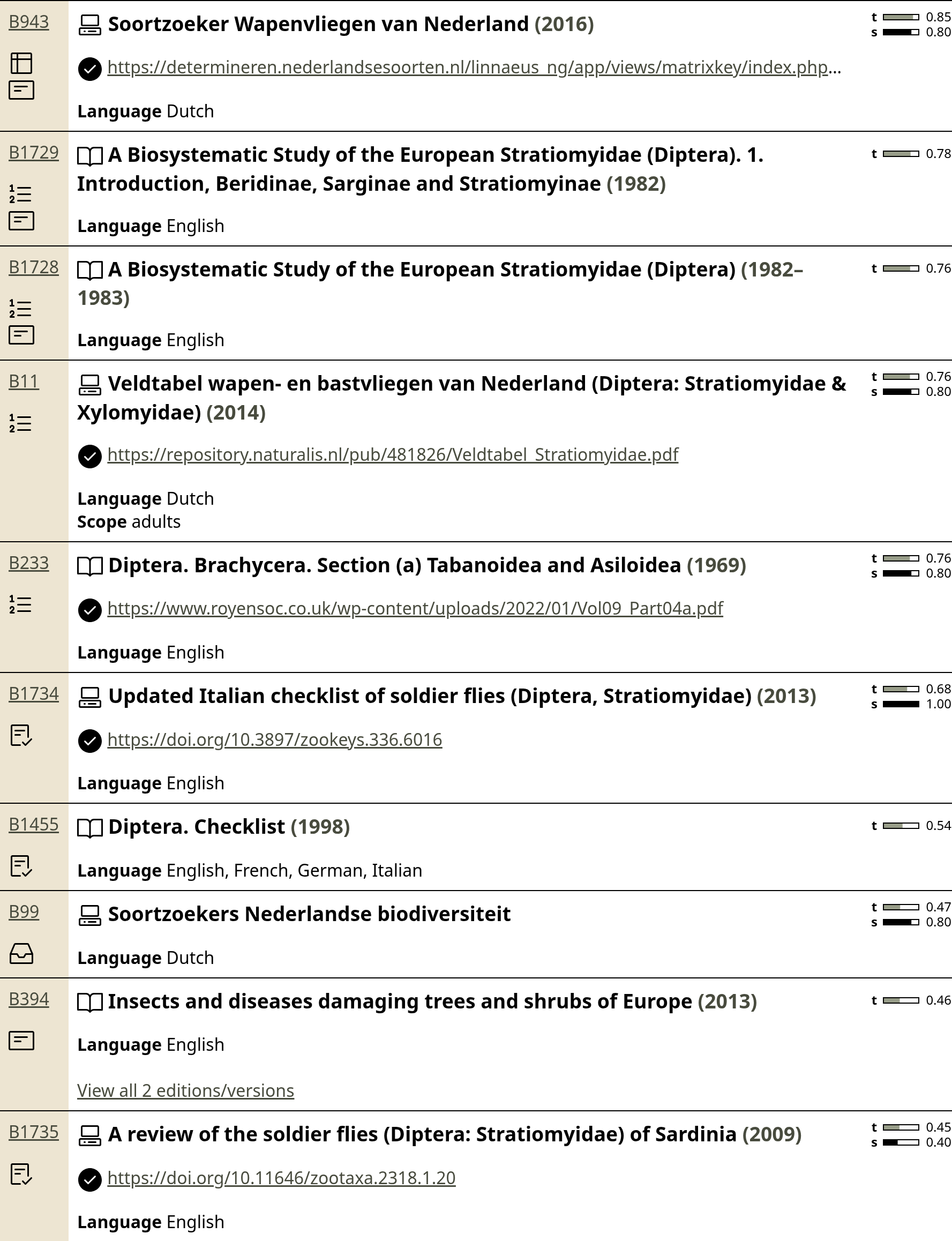

Supplement: Supplementary material 4 — Figure S2 [file bdj-13-e161726-s004.png]
